# Health promotion and disease prevention with digital technologies for older people: Scoping review

**DOI:** 10.1093/eurpub/ckac131.166

**Published:** 2022-10-25

**Authors:** KK De Santis, L Mergenthal, L Christianson, H Zeeb

**Affiliations:** 1 Department of Prevention and Evaluation, Leibniz Institute BIPS, Bremen, Germany; 2 Leibniz-Science Campus Digital Public Health, Bremen, Germany; 3 Department of Administration, Leibniz Institute BIPS, Bremen, Germany; 4 Faculty 11 Human and Health Sciences, University of Bremen, Bremen, Germany

## Abstract

**Background:**

In the aging world digital technologies are needed to target the health needs of older people. This study aimed to identify digital technologies for health promotion and disease prevention for older people by performing a scoping review.

**Methods:**

A search of MEDLINE, PsycINFO, CINAHL and SCOPUS on 09.03.2022 identified 2150 studies. The inclusion criteria were: 1) Population: older people, 2) Concept: any digital health technology, 3) Context: health promotion and disease prevention in home or community settings. Preliminary study selection was performed automatically using the smart groups function in EndNote. Studies were clustered by digital technology type, health target, study design and study focus. Final study selection and data coding will be performed manually by two authors.

**Results:**

Of the 2150 studies, 1874 studies were excluded, 159 studies met the inclusion criteria 1) and 2), but addressed different contexts (digital competence, digital technology development, disease management) or setting (care) and 117 studies were included. Digital technology types were: 1) any technologies (digital, virtual, video, eHealth or telehealth), 2) internet websites accessed via computer, 3) SMS or mobile phones, 4) exergaming or 5) smartphones or wearables. Health targets were: 1) physical activity, 2) mental health and wellness, 3) nutrition or 4) cognitive functioning. Study designs included primary studies (randomized-controlled trials) or reviews (systematic or scoping). Study focus was on effectiveness, feasibility or evaluation of digital technologies.

**Conclusions:**

The health needs of older people are addressed by older technologies. Newer studies use heterogeneous terminology when referring to digital technologies. Future studies should focus on multiple aspects of healthy aging beyond mobility. More work is also needed to understand if and how a shift towards newer technologies occurs and if that is associated with health benefits in older people.

**Key messages:**

• Older digital technologies (computers and mobile phones) address the health needs of older people.

• Mobility is the main health target of digital technologies for older people in the context of health promotion and disease prevention.

